# Exoproteome analysis of *Clostridium cellulovorans* in natural soft-biomass degradation

**DOI:** 10.1186/s13568-014-0089-9

**Published:** 2015-01-24

**Authors:** Kohei Esaka, Shunsuke Aburaya, Hironobu Morisaka, Kouichi Kuroda, Mitsuyoshi Ueda

**Affiliations:** Division of Applied Life Sciences, Graduate School of Agriculture, Kyoto University, Sakyo-ku, Kyoto Japan; Kyoto Integrated Science and Technology Bio-Analysis Center, Shimogyo-ku, Kyoto Japan

**Keywords:** *Clostridium cellulovorans*, Cellulosome, Soft-biomass degradation, Proteome analysis, Monolithic column

## Abstract

*Clostridium cellulovorans* is an anaerobic, cellulolytic bacterium, capable of effectively degrading various types of soft biomass. Its excellent capacity for degradation results from optimization of the composition of the protein complex (cellulosome) and production of non-cellulosomal proteins according to the type of substrates. In this study, we performed a quantitative proteome analysis to determine changes in the extracellular proteins produced by *C. cellulovorans* for degradation of several types of natural soft biomass. *C. cellulovorans* was cultured in media containing bagasse, corn germ, rice straw (natural soft biomass), or cellobiose (control). Using an isobaric tag method and a liquid chromatograph equipped with a long monolithic silica capillary column/mass spectrometer, we identified 372 proteins in the culture supernatant. Of these, we focused on 77 saccharification-related proteins of both cellulosomal and non-cellulosomal origins. Statistical analysis showed that 18 of the proteins were specifically produced during degradation of types of natural soft biomass. Interestingly, the protein Clocel_3197 was found and commonly involved in the degradation of every natural soft biomass studied. This protein may perform functions, in addition to its known metabolic functions, that contribute to effective degradation of natural soft biomass.

**Electronic supplementary material:**

The online version of this article (doi:10.1186/s13568-014-0089-9) contains supplementary material, which is available to authorized users.

## Introduction

Cellulosic and herbaceous types of biomass (soft biomass) such as rice straw, switchgrass, and bagasse show promise as substrates for the production of chemical products and fuels. However, it is difficult to degrade soft biomass (Lynd et al. 1999). Cellulose is comprised of a glucose-linked structure that is resistant to degradation due to the number of hydrogen bonds in its crystalline structure (Mansfield et al. 1999). Chemical procedures, including processing with strong acids, high pressures, or high temperatures, are generally employed to degrade cellulose to glucose; however, these methods impose an environmental burden. In addition, degradation strategies must be optimized according to the type of soft biomass, based on individual structures and components.

The artificial and commercial cellulase cocktails currently available are expensive; however, several naturally occurring microbes present an attractive alternative. We have focused on the cellulolytic bacterium *Clostridium cellulovorans. C. cellulovorans* is a mesophilic, anaerobic bacterium that can degrade various components of plant cell walls, including not only cellulose, but also hemicelluloses and pectin (Sleat et al. 1984). Previously, we performed genome analysis of *C. cellulovorans* and demonstrated that it produced a “cellulosome” (Tamaru et al. 2010), a multi-enzyme complex that is known to be produced by several types of cellulolytic and anaerobic bacteria (Bae et al. 2013; Bayer et al. 2004; Doi and Kosugi 2004) such as *C. thermocellum* (Lamed et al. 1983b; Bayer et al. 1983; Lamed et al. 1983a) and *C. cellulolyticum* (Desvaux 2005; Gal et al. 1997). *C. cellulovorans* has high cellulolytic activity due to the presence of numerous polysaccharide degradation-related proteins that show synergistic effects (Fierobe et al. 2002). Genomic analysis of *C. cellulovorans* indicated the presence of 57 cellulosome-related genes, including four scaffold and 53 cellulosomal protein-encoding genes (Tamaru et al. 2010). The major scaffold protein, CbpA, is composed of nine cohesin domains that bind to various cellulosomal proteins (Tamaru 2001). Using proteome analysis, we reported that *C. cellulovorans* optimized the composition of its cellulosomal protein according to the type of basal substrates (cellobiose, avicel, and xylan) (Morisaka et al. 2012), and that this ability played a major role in polysaccharide degradation (Matsui et al. 2013). However, compared to the genomes of other cellulosome-producing clostridial species, the genome of *C. cellulovorans* contains a very large number (190) of non-cellulosomal protein-encoding genes (Tamaru et al. 2011). Non-cellulosomal proteins do not form a complex (cellulosome) and function as free saccharification-related enzymes. We reported that non-cellulosomal proteins also played a key role in effective degradation of basal biomass (Matsui et al. 2013). *C. cellulovorans* could effectively degrade various types of natural soft biomass via the cooperative activity of cellulosomal and non-cellulosomal proteins. This unique and cooperative feature offers the potential to enhance the efficiency of soft-biomass degradation. However, few comprehensive and molecular studies of the degradation of natural soft biomass have been reported. To improve the efficiency of soft-biomass utilization, it will be useful to study the changes in the *C. cellulovorans* protein profile in response to various types of natural soft biomass.

In this study, we performed a quantitative analysis of the cellulosomal and non-cellulosomal proteins produced by *C. cellulovorans* during the degradation of several types of natural soft biomass. We used bagasse (the byproduct of sugar cane processing), corn germ (corn embryos), and rice straw as carbon sources. Proteins in the culture supernatant (exoproteome) were analyzed using a LC-MS/MS system equipped with a long monolithic silica capillary column (470 cm), as described previously (Matsui et al. 2013; Morisaka et al. 2012). We identified the individual protein profiles of the exoproteomes, including both cellulosomal and non-cellulosomal proteins. Additionally, integrated proteome and genome analysis indicated that *C. cellulovorans* produced proteins that showed promise for improving the efficiency of degradation of natural soft biomass.

## Methods

### Cell culture and medium

*C. cellulovorans* 743B (ATCC35296) was grown anaerobically as previously described (Sleat et al. 1984), differing only in carbon source, which was replaced by 0.3% (w/v) cellobiose and 0.3% (w/v) soft biomass.

### Growth substrates

Cellobiose (Sigma, St Louis, MO, USA) and cellulosic soft biomass were used in the growth experiments. Bagasse, corn germ, and rice straw were used as soft biomass. Bagasse, containing 39.6% cellulose, 20.2% hemicellulose, 25.9% lignin, 14.3% other components, was provided by H. Nonaka, Mie University (Nonaka et al. 2013; Ren and Funaoka 2009); corn germ, containing 10.9% cellulose, 23.3% hemicellulose, 0.6% lignin, 65.2% other components, was provided by Tsuji Oil Mill Co. Ltd (Furuya et al. 2010); and rice straw (Nakanishi et al. 2012), containing 39.2% cellulose, 27.4% hemicellulose, 4.4% lignin, 29.0% other components, was provided by H. Miyake, Mie University. These were crushed for 1 min by using a Hi-Power Blender MX1100XTS (Waring Commercial, Torrington, CT, USA), and the resulting soft biomass (diameter < 250 μm) was used for *C. cellulovorans* culture.

### Preparation of extracellular proteins (exoproteome) for quantitative proteome analysis

Samples from *C. cellulovorans* cultures were prepared for proteome analysis as previously described (Matsui et al. 2013). Each stationary-phase culture (50 mL) was centrifuged (6,000 × *g*, 25°C), and the supernatant was subjected to ultrafiltration using an Amicon Ultra-15 Centrifugal Filter Unit (MWCO 10 kDa, Millipore, Darmstadt, Hessen, Germany) to obtain the extracellular proteins. The concentrated samples were independently dissolved in 100 μL of triethylammonium bicarbonate buffer (200 mM), to which 5 μL of Tris(2-carboxyethyl)phosphine (200 mM) was added, and the reaction was allowed to proceed for 60 min at 55°C. To this mixture, 5 μL of iodoacetamide (375 mM) was added, and the reaction continued for 30 min, protected from light, at room temperature. Sequencing grade modified trypsin (1 μg/μL; Promega, Madison, WI, USA) was added (2 μL), and the proteins were digested overnight at 37°C. The four proteome samples (cellobiose, bagasse, corn germ, and rice straw) were labeled using a tandem mass tag (TMT) 6-plex labeling kit (Thermo Fisher Scientific, Waltham, MA, USA) with reporters at *m/z* = 128, 129, 130, and 131, respectively, in 41 μL acetonitrile. After 60 min of reaction at room temperature, 8 μL of 5% (w/v) hydroxylamine was added to each tube and mixed for 15 min. In addition, a mixture of tryptic fragments from all substrates was combined with TMT-126 (reporter at *m/z* = 126) as an internal standard for quantification. The aliquots were then pooled and evaporated under vacuum and dissolved in 100 μL of trifluoroacetic acid (0.1%) and used for LC-MS/MS analysis.

### Exoproteome analysis

Proteome analysis was performed using an LC (Ultimate 3000®; Thermo Fisher Scientific)-MS/MS (LTQ Orbitrap Velos Mass Spectrometer®; Thermo Fisher Scientific) system equipped with a long monolithic column, as previously described (Matsui et al. 2013; Morisaka et al. 2012). Tryptic digests were separated by reversed-phase chromatography using a monolithic silica capillary column (470 cm long, 0.1 mm ID), at a flow rate of 500 nL/min. The gradient was provided by changing the mixing ratio of the two eluents: A, 0.1% (v/v) formic acid and B, 80% acetonitrile containing 0.1% (v/v) formic acid. The gradient was started with 5% B, increased to 45% B for 600 min, further increased to 95% B to wash the column, returned to the initial condition, and held for re-equilibration. The separated analytes were detected using a mass spectrometer with a full scan range of 350–1,500 *m/z* (resolution 60,000), followed by 10 data-dependent higher-energy c-trap dissociation (HCD) MS/MS scans acquired for TMT reporter ions, using 40% normalized collision energy in HCD with 0.1 ms activation time and an electrospray ionization (ESI) voltage of 2.3 kV. The ion transfer tube temperature was set to 280°C. Triplicate analyses were performed for each sample in three independent experiments, and the collected data were reviewed for protein identification and quantification.

Data analysis was performed using Proteome Discoverer software (Thermo Fisher Scientific). Protein identification was performed using the Mascot algorithm against the *C. cellulovorans* protein database (4,254 sequences) from NCBI (National Center for Biotechnology Information, http://www.ncbi.nlm.nih.gov/), with a precursor mass tolerance of 20 ppm and a fragment ion mass tolerance of 50 mmu. Carbamidomethylation of cysteine and a TMT 6-plex at the N-terminus were set as fixed modifications. Protein quantification was performed using the Reporter Ions Quantifier with the TMT 6-plex method. The data were then filtered with a cut-off criteria of *q*-value ≤ 0.05, corresponding to a 5% false discovery rate (FDR) on a spectral level. The values for the exponentially modified protein abundance index (emPAI) (Ishihama et al. 2005) were used to estimate the abundance of cellulosomal and non-cellulosomal proteins. Proteins with no missing values in three replicates were accepted in the protein quantification analysis. Global median normalization was performed to normalize the quantity of each tryptic digest injected into the mass spectrometer.

## Results

### Analysis of proteins in *C. cellulovorans* culture supernatants

**Figure 1:**
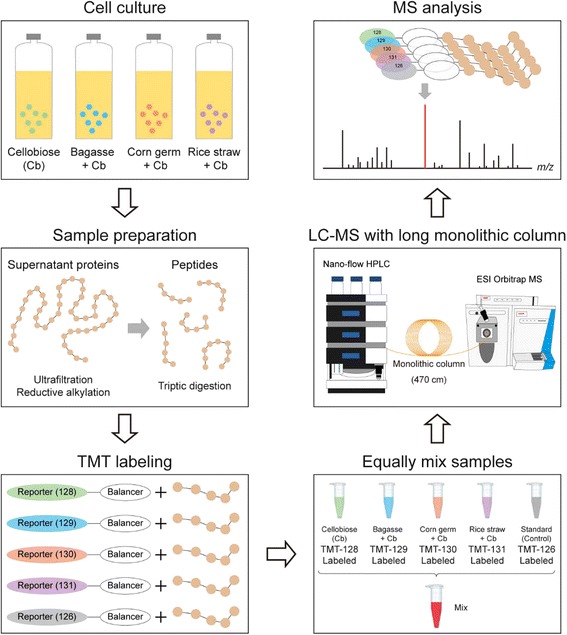
**Experimental procedure for** ***C. cellulovorans*** **exoproteome analysis.** Proteome analysis was performed as previously described (Matsui et al. 2013). Proteins in the culture supernatant of *C. cellulovorans* grown in the presence of cellobiose (Cb), bagasse, corn germ, or rice straw were individually reductive-alkylated and digested with trypsin, and tryptic fragments were labeled with tandem mass tags (TMTs). The labeled peptides were mixed and injected into the LC-MS/MS system with a long monolithic column for mass measurement, and the data collected were used for protein quantification.

**Figure 2:**
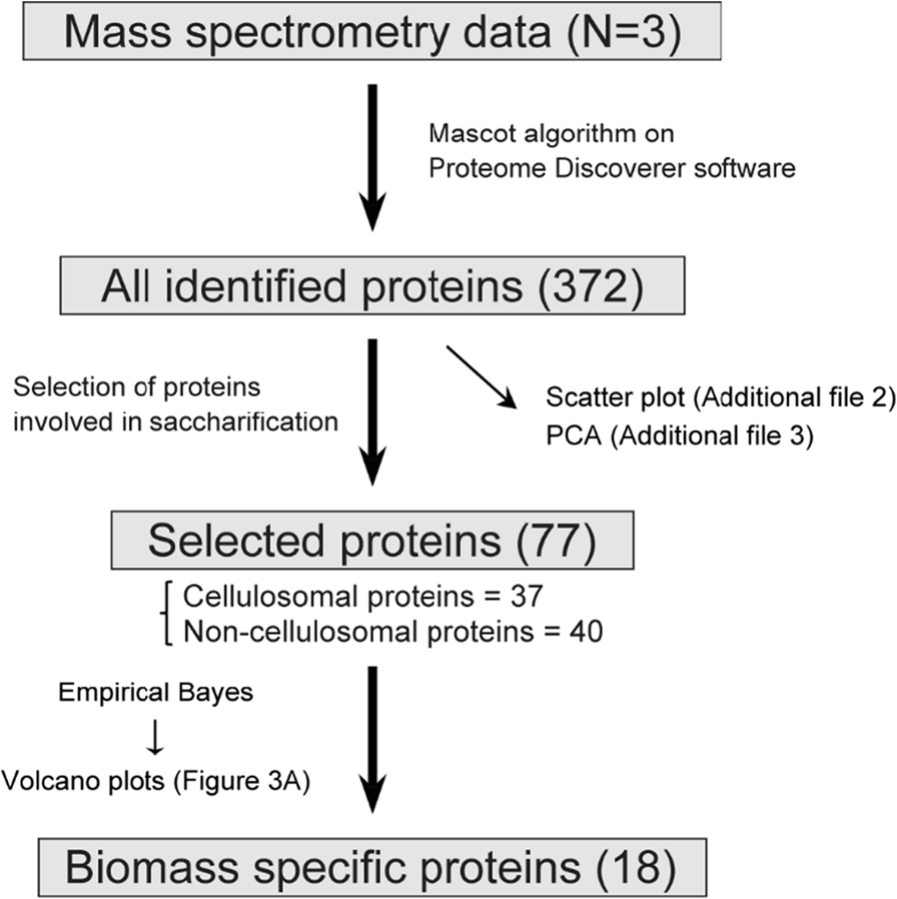
**Statistical analysis for detection of proteins specific for degradation of each natural soft biomass type.** The total number of proteins identified and the number of proteins involved in saccharification are shown. A total of 372 proteins were initially quantified. To confirm reproducibility, scatter plots of the quantitative values were created, and principal component analysis was performed (Additional files 2, 3). For detection of specific proteins for natural soft biomass degradation, 77 cellulosomal and non-cellulosomal proteins were selected from the 372 proteins identified. To determine which proteins were biomass-specific, empirical Bayes moderated *t*-tests were performed and volcano plots were generated (Figure 3A).

To investigate the degradation of natural soft biomass, proteins were isolated from the supernatant of stationary-phase *C. cellulovorans* cultures grown on bagasse, corn germ, or rice straw, and subjected to LC-MS/MS analysis (Figure 1) (Matsui et al. 2013). The mass spectrometry data collected were used for exoproteome analysis, as shown in Figure 2.

**Figure 3:**
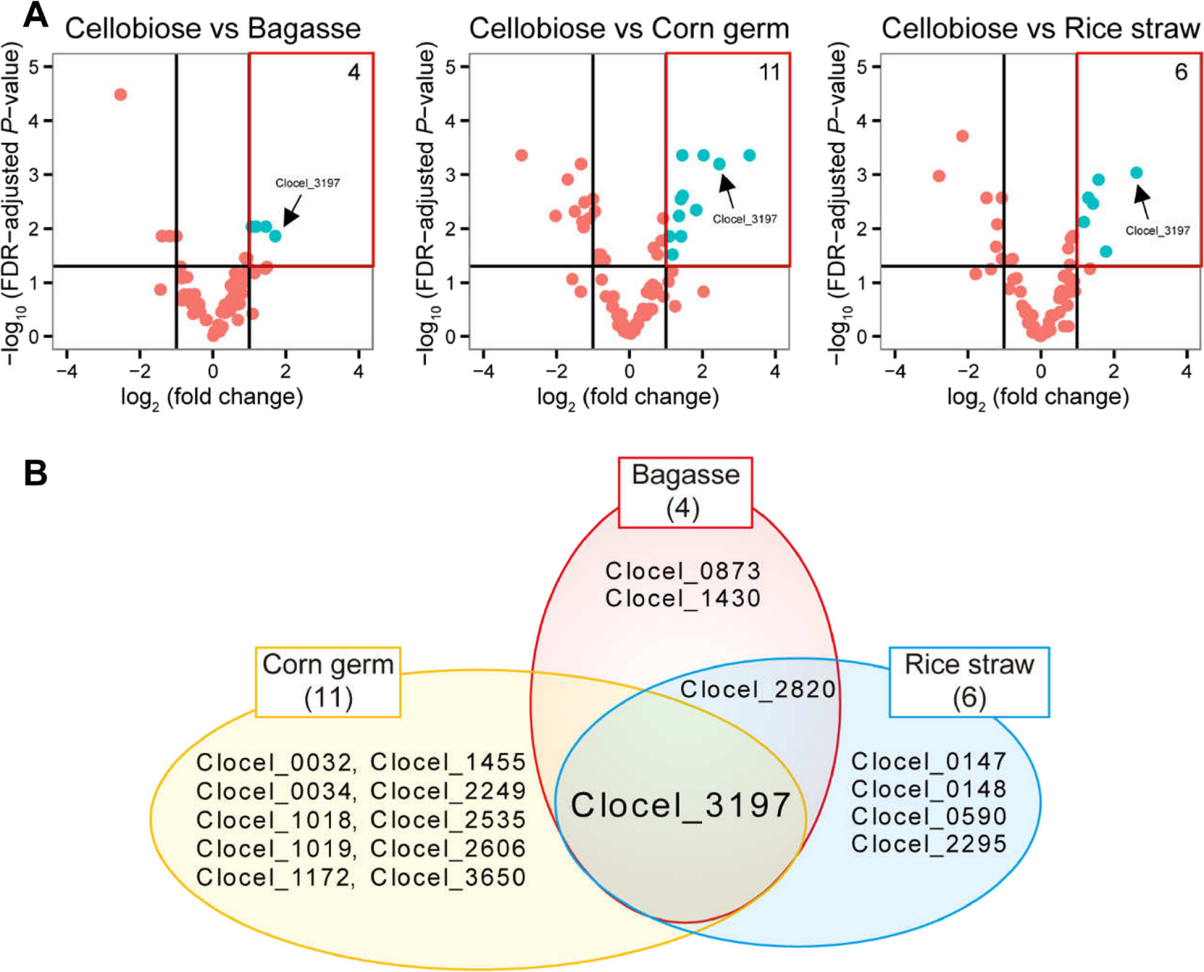
**Identification of biomass-specific proteins.** Changes in protein ratios in the exoproteome are shown as volcano plots for each type of biomass versus cellobiose **(A)**. Extracellular proteins were present in different amounts and were identified as being secreted in the presence of a specific type of biomass. These showed an FDR-adjusted *P*-value of < 0.05 and a fold change in protein ratio of > 2 compared to cellobiose; these are indicated here as blue dots. Thus, four bagasse-, 11 corn germ-, and six rice straw-specific proteins were identified, and they are shown in the Venn diagram **(B)**.

To normalize the amount of each tryptic digest injected into the mass spectrometer, the identified proteins were standardized with the median. To determine which proteins were differentially produced for each type of soft biomass, we focused on 77 cellulosomal and non-cellulosomal saccharification-related enzymes, chosen from among the 372 proteins identified (Additional file 1). Next, we performed empirical Bayes moderated *t*-tests and created volcano plots (Figure 3A) using the quantitative values for each of the 77 proteins (Matsui et al. 2013); proteins produced in culture with cellobiose were used as controls. *P*-values were adjusted using the Benjamini-Hochberg method to avoid the problem of multiple testing. Proteins which met the criteria (FDR-adjusted *P*-value < 0.05 and fold-change of protein ratio > 2 as compared to cellobiose) were defined as individual “biomass-specific proteins.” Four bagasse-, 11 corn germ-, and six rice straw-specific proteins were identified (Figure 3B and Table 1).

**Table 1:** **The list of biomass-specific proteins**

**Biomass specifically**	**Protein type**	**Locus**	**Name** ^**a**^	**CAZy family(ies)** ^**b**^	**vs Cellobiose**		**emPAI** ^**d**^
					**log**_**2**_ **-fold change**	**FDR-adjusted** ***P-*** **value** ^**c**^	
Bagasse (4)	Cellulosomal	Clocel_2820	HbpA	NA	1.45	9.13E-03	2.49
	Non-cellulosomal	Clocel_1430		GH31	1.19	9.13E-03	0.55
		Clocel_3197		GH130	1.71	1.37E-02	0.79
		Clocel_0873		PL9	1.07	9.13E-03	1.19
Corn germ (11)	Cellulosomal	Clocel_3650		GH44	1.45	4.39E-04	0.53
	Non-cellulosomal	Clocel_1018		GH1	1.41	2.85E-03	0.77
		Clocel_1019		GH1	3.29	4.39E-04	0.34
		Clocel_2606		GH5,CBM46	1.36	5.78E-03	2.44
		Clocel_0034		GH31	1.46	2.48E-03	0.51
		Clocel_2535		GH43	1.09	1.38E-02	0.20
		Clocel_1455		GH53,CBM61,CBM61,CBM61	2.03	4.39E-04	0.13
		Clocel_0032		GH94	1.83	4.50E-03	0.36
		Clocel_3197		GH130	2.46	6.34E-04	0.79
		Clocel_1172		PL1	1.41	1.38E-02	0.50
		Clocel_2249		NA	1.18	3.00E-02	1.01
Rice straw (6)	Cellulosomal	Clocel_2295	XynA	GH11,CE4	1.79	2.64E-02	0.69
		Clocel_0148		NA	1.59	1.24E-03	0.65
		Clocel_0147	CpiA	NA	1.3	2.72E-03	9.10
		Clocel_2820	HbpA	NA	1.43	3.46E-03	2.49
	Non-cellulosomal	Clocel_3197		GH130	2.63	9.16E-04	0.79
		Clocel_0590		NA	1.19	7.51E-03	5.27

^a^Name: Names of only the reported proteins were shown.

^b^See http://www.cazy.org/. NA, not annotated (not included in CAZy database).

^c^ *P*-values were adjusted for multiple testing with the Benjamini-Hochberg method.

^d^emPAI: the values of exponentially modified protein abundance index.

### Bagasse-specific proteins

Four bagasse-specific proteins were identified, including one cellulosomal and three non-cellulosomal proteins. The cellulosomal protein was Clocel_2820 (HbpA), a scaffolding protein with no enzymatic activity, similar to CbpA, which is the main scaffolding protein of the cellulosome. HbpA has been reported to enhance cellulosomal cellulase (Clocel_1150 (EngB) or Clocel_2819 (EngL)) activity on solid substrates such as avicel or corn fiber, but not on soluble carboxymethyl cellulose (Matsuoka et al. 2007). Thus, *C. cellulovorans* might increase the production of HbpA to accelerate cellulase activity on solid biomass. Among the non-cellulosomal proteins, Clocel_0873, which is classified as a member of the PL9 family by the Carbohydrate-Active enZymes (CAZy) database (Lombard et al. 2014), is considered to have pectate lyase activity. Clocel_1430, classified as a member of the GH31 family, is considered to have α-xylosidase activity. Clocel_0873 is a pectin-specific protein, and Clocel_1430 is a xylan-specific protein (Matsui et al. 2013). *C. cellulovorans* might recognize the pectin contained in bagasse and produce these proteins in response. Clocel_3197, a member of the GH130 family, was also detected.

### Corn germ-specific proteins

One cellulosomal protein, Clocel_3650, and 10 non-cellulosomal proteins were identified. Clocel_3650, a member of the GH44 family, is considered to have endoglucanase activity. Among the non-cellulosomal proteins, Clocel_2606, classified as a member of the GH5 and CBM46 families, is considered to have cellulose-binding as well as cellulose-degrading activities (Aspeborg et al. 2012); it is a phosphoric acid swollen cellulose (PASC)-specific protein (Matsui et al. 2013). Clocel_3650 and Clocel_2606 are considered to be involved in acceleration of cellulose degradation. Clocel_0034, a member of the GH31 family, is considered to have α-xylosidase activity, while Clocel_2535, classified as a member of the GH43 family, is considered to have β-xylosidase activity. These xylan degradation-related proteins are thought to contribute to the acceleration of xylan degradation. Clocel_1455, a member of the GH53 and CBM61 families, is considered to have endo-1, 4-β-galactosidase activity and 1, 4-β-galactan binding ability (Cid et al. 2010). Clocel_0032 is also a PASC-specific protein (Matsui et al. 2013). It is a member of the GH94 family and is considered to have cellodextrin phosphorylase activity. Clocel_1172 is a pectin-specific protein (Matsui et al. 2013). It is a member of the PL1 family and is thought to have pectate lyase activity. Clocel_2249 is considered to be a glucuronate isomerase, and it also contributes to the acceleration of pectin degradation. Clocel_1018 and Clocel_1019 are members of the GH1 family; enzymes in this family are thought to have various enzymatic activities, such as β-glucosidase and β-xylosidase activities. Clocel_3197, which was identified among the bagasse-specific proteins, was also detected.

### Rice straw-specific proteins

The rice straw-specific proteins consisted of four cellulosomal and two non-cellulosomal proteins. Among the cellulosomal proteins, Clocel_2295 (XynA), a member of the GH11 and CE4 families, has endoxylanase and acetyl-xylan esterase activities (Kosugi et al. 2002) and is a xylan-specific protein (Matsui et al. 2013). Since xylan (xylose) is abundant in the hemicellulose of rice straw (Yoswathana et al. 2010), *C. cellulovorans* is considered to recognize xylose and increase the production of XynA for effective degradation of rice straw. Clocel_0147 is a cysteine protease inhibitor (Meguro et al. 2011), which is considered to protect microbe and their cellulosomes from plant protease attack. Our results suggest that *C. cellulovorans* recognizes rice as its substrate and produces large amounts of cyspin as a defense mechanism. Clocel_0148, a protein of unknown function, was identified as a rice straw-specific protein. Clocel_2820 (HbpA), found among the bagasse-specific proteins, was also detected. Clocel_0590, detected among the non-cellulosomal proteins, is thought to be a xylose isomerase (Ota et al. 2013) and to be involved in degradation of the xylan contained in rice straw. Clocel_3197, found in both the bagasse- and corn germ-specific proteins, was also detected.

## Discussion

We identified several proteins involved in degradation of various types of biomass; analysis of replicates showed that the results were reproducible. Scatter plots of normalized quantitative values for all combinations showed high correlation factors (Additional file 2). Principal component analysis (PCA) was performed for all 372 identified proteins to confirm that the proteome profile was similar between three biological replicates. The PCA score plots (Additional file 3) showed a degree of high similarity between biological replicates, and the plots for each substrate formed individual groups. These results indicate that the quantitative proteome analysis showed a high degree of reproducibility and reliability.

Of the total 372 proteins identified, 77 proteins were determined to be involved in saccharification. Of these, 37 were cellulosomal proteins and 40 were non-cellulosomal proteins (Additional file 1). *C. cellulovorans* possesses 57 cellulosomal protein-encoding genes and 190 non-cellulosomal protein-encoding genes. Therefore, *C. cellulovorans* produced 64.9% (37 of 57) of its cellulosomal proteins and 21.1% (40 of 190) of its non-cellulosomal proteins for degradation of the types of natural soft biomass examined here.

From the statistical analysis, Clocel_3197 was commonly identified as all biomass-specific proteins in *C. cellulovorans* exoproteome. Interestingly, SignalP analysis did not detect a signal peptide-encoding sequence for Clocel_3197. Clocel_3197 is likely to be localized to the exterior of cells, based on the emPAI of Clocel_3197, which was nearly equal to that of Clocel_2295 (XynA) (Table 1), which has a signal peptide-encoding sequence. This result also indicated that sample preparation did not lyse cells. Clocel_3197, a member of the GH130 family, has been annotated as a d-fructose 4-*o*-β-d-mannosyl-d-glucose phosphorylase. Some species, such as *Bacteroides fragilis*, have the homologue of Clocel_3197 containing operon and it plays an important role in mannan catabolic pathway (Senoura et al. 2011). However, all enzymes related to mannan degradation or metabolism were not detected (Additional file 1). Thus, this protein may also possess an extracellular function. Previous reports have indicated the presence of several proteins lacking signal peptides that are secreted by unconventional pathways (López-Villar et al. 2006; Kinseth et al. 2007). For example, several metabolites produced by Clocel_3197, which have different functions like those observed in moon-lighting proteins (Kinseth et al. 2007; López-Villar et al. 2006) may play an important role during substrate recognition and natural soft-biomass degradation by *C. cellulovorans*. The mechanisms of substrate degradation and recognition remain unknown, and this protein may be useful in future investigations of the substrate degradation and recognition mechanisms of *C. cellulovorans*.

In conclusion, we quantified the cellulosomal and non-cellulosomal protein profiles produced by *C. cellulovorans* cultured on various types of soft biomass. A total of 77 cellulosomal and non-cellulosomal proteins were identified from the *C. cellulovorans* culture supernatant by using an LC-MS/MS system equipped with a long monolithic silica capillary column. Empirical Bayes moderated *t*-tests and volcano plots identified four bagasse-, 11 corn germ-, and six rice straw-specific proteins. Clocel_3197 was identified from the supernatant of cultures grown on all three types of biomass, and may perform as-yet-unknown functions that contribute to effective degradation of natural soft biomass.

## Additional files

Additional file 1:
**The 372 proteins identified.** Proteome analytes were injected to LC-MS/MS system. Collected data were used for protein identification by Proteome Discoverer software. Three independent biological experiments were performed, and proteins identified in every replicates with a number of used peptides per protein (≥3) were accepted. As a result, 372 proteins were successfully identified. Additional file 2:
**Scatter plots of the three biological replicates for each substrate.** The fold-change values of identified 372 proteins (Additional file 1) by using the Reporter Ions Quantifier with the TMT 6-plex method were normalized using global median. Scatter plots of normalized values were depicted using the data derived from three biological replicates of each culture (cellobiose, bagasse, corn germ, and rice straw). The values of Pearson’s correlation were successfully high in each combination. Additional file 3:
**Principal component analysis of the data from the three biological replicates.** Principal component analysis was performed using normalized fold-change values of identified 372 proteins (Additional file 1) for investigation of the similarity of protein production profile between each biological replicate. Proteome data from each substrate clustered in close proximity. The cumulative contribution rate for principal component (PC) PC1 to PC3 was 87.5% (green: cellobiose; red: bagasse; purple: corn germ; blue: rice straw).

## Abbreviations

ATCC: American type culture collection CAZy, carbohydrate-active enZymes
Cb: Cellobiose
CBM: Carbohydrate-binding modules
CE: Carbohydrate esterases
Cyspin: Cystein protease inhibitor
emPAI: Exponentially modified protein abundance index
ESI: Electrospray ionization
FDR: False discovery rate
GH: Glycoside hydrolases
GT: Glycosyl transferases
HCD: Higher-energy c-trap dissociation
HPLC: High performance liquid chromatography
ID: Internal diameter
LC-MS: Liquid chromatography-mass spectrometry
NCBI: National Center for Biotechnology Information
PCA: Principal component analysis
PL: Polysaccharide lyases
PMI: Phosphomannose isomerase
PASC: Phosphoric acid swollen cellulose
SDS: Sodium sodecyl sulfate
TMT: Tandem mass tag
