# Supplementary material for: Exoproteome analysis of Clostridium cellulovorans in natural soft-biomass degradation
Source: AMB Express. 2015 Jan 24;5:2. doi: 10.1186/s13568-014-0089-9 (PMC4305082; doi:10.1186/s13568-014-0089-9)
Supplement: Additional file 1: — The 372 proteins identified. Proteome analytes were injected to LC-MS/MS system. Collected data were used for protein identification by Proteome Discoverer software. Three independent biological experiments were performed, and proteins identified in every replicates with a number of used peptides per protein (≥3) were accepted. As a result, 372 proteins were successfully identified. [file 13568_2014_89_MOESM1_ESM.pdf]

Table S1 The list of 372 identified proteins

Protein type	Accession	Locus	Description
Cellulosomal (37)	gi302578426	Clocel_2741	glycoside hydrolase family protein
	gi302578297	Clocel_2600	hypothetical protein Clocel_2600
	gi302579308	Clocel_3650	Dockerin type 1
	gi302576686	Clocel_0930	glycoside hydrolase family protein
	gi302578276	Clocel_2576	glycoside hydrolase family protein
	gi302576737	Clocel_0983	glycoside hydrolase family protein
	gi302578789	Clocel_3111	glycoside hydrolase family protein
	gi302576378	Clocel_0619	glycoside hydrolase family protein
	gi302576894	Clocel_1150	glycoside hydrolase family protein
	gi302579026	Clocel_3359	cellulase
	gi302578506	Clocel_2822	glycoside hydrolase family protein
	gi302578505	Clocel_2821	glycoside hydrolase family protein
	gi302578503	Clocel_2819	glycoside hydrolase family protein
	gi302578501	Clocel_2816	glycoside hydrolase family protein
	gi302577358	Clocel_1624	glycoside hydrolase family protein
	gi302578502	Clocel_2818	Dockerin type 1
	gi302579768	Clocel_4119	Dockerin type 1
	gi302578302	Clocel_2607	Dockerin type 1
	gi302578275	Clocel_2575	Dockerin type 1
	gi302578010	Clocel_2295	glycoside hydrolase family protein
	gi302578583	Clocel_2900	glycoside hydrolase family protein
	gi302578507	Clocel_2823	glycoside hydrolase family protein
	gi302577357	Clocel_1623	Pectate lyase/Amb allergen
	gi302577166	Clocel_1425	Ig domain-containing protein
	gi302575925	Clocel_0148	Ig domain-containing protein
	gi302576230	Clocel_0466	Dockerin type 1
	gi302575924	Clocel_0147	Proteinase inhibitor I42, chagasin
	gi302575923	Clocel_0146	Proteinase inhibitor I42, chagasin
	gi302576736	Clocel_0982	Kelch repeat type 1-containing protein
	gi302579275	Clocel_3615	hypothetical protein Clocel_3615
	gi302578867	Clocel_3193	Dockerin type 1
	gi302576926	Clocel_1182	hypothetical protein Clocel_1182
	gi302576925	Clocel_1181	hypothetical protein Clocel_1181
	gi302578790	Clocel_3112	hypothetical protein Clocel_3112
	gi302577968	Clocel_2240	hypothetical protein Clocel_2240
	gi302578508	Clocel_2824	cellulosome anchoring protein cohesin subunit
	gi302578504	Clocel_2820	cellulosome anchoring protein cohesin subunit
Non-cellulosomal (40)	gi302576770	Clocel_1018	glycoside hydrolase family 1
	gi302576771	Clocel_1019	glycoside hydrolase family 1
	gi302578565	Clocel_2882	6-phospho-beta-glucosidase
	gi302579733	Clocel_4083	glycoside hydrolase family 2
	gi302576819	Clocel_1072	glycoside hydrolase family protein
	gi302576670	Clocel_0912	cellulase
	gi302578301	Clocel_2606	LPXTG-motif cell wall anchor domain-containing protein
	gi302578916	Clocel_3242	glycoside hydrolase family protein
	gi302579320	Clocel_3662	cellulase
	gi302577161	Clocel_1420	LPXTG-motif cell wall anchor domain-containing protein
	gi302577217	Clocel_1478	glycoside hydrolase family protein
	gi302576879	Clocel_1134	LPXTG-motif cell wall anchor domain-containing protein
	gi302577171	Clocel_1430	glycoside hydrolase family protein
	gi302575811	Clocel_0034	glycoside hydrolase family protein

	gi302578485	Clocel_2800	Alpha-galactosidase
	gi302578211	Clocel_2511	glycoside hydrolase clan GH-D
	gi302576321	Clocel_0560	Alpha-galactosidase
	gi302575818	Clocel_0041	beta-galactosidase
	gi302578235	Clocel_2535	LPXTG-motif cell wall anchor domain-containing protein
	gi302579315	Clocel_3657	xylan 1,4-beta-xylosidase
	gi302577751	Clocel_2020	alpha-L-arabinofuranosidase
	gi302577196	Clocel_1455	LPXTG-motif cell wall anchor domain-containing protein
	gi302575809	Clocel_0032	glycosyltransferase
	gi302576159	Clocel_0391	glycosyltransferase
	gi302578871	Clocel_3197	glycosidase-like protein
	gi302578870	Clocel_3196	glycosidase-like protein
	gi302576916	Clocel_1172	Pectate lyase/Amb allergen
	gi302576763	Clocel_1011	Pectate lyase/Amb allergen
	gi302576631	Clocel_0873	pectate lyase/Amb allergen
	gi302579492	Clocel_3834	pectate lyase
	gi302578791	Clocel_3113	pectate lyase
	gi302575946	Clocel_0171	G-D-S-L family lipolytic protein
	gi302576735	Clocel_0981	Ig domain-containing protein
	gi302576349	Clocel_0590	xylose isomerase
	gi302576997	Clocel_1254	L-arabinose isomerase
	gi302577965	Clocel_2237	L-fucose isomerase
	gi302577976	Clocel_2249	glucuronate isomerase
	gi302577989	Clocel_2263	4-deoxy-L-threo-5-hexosulose-uronate ketol-isomerase
	gi302578500	Clocel_2815	Carbohydrate-binding CenC domain-containing protein
	gi302579721	Clocel_4071	peptidoglycan glycosyltransferase
Others (295)	gi302577416	Clocel_1684	pyruvate ferredoxin/flavodoxin oxidoreductase
	gi302577026	Clocel_1284	Glu/Leu/Phe/Val dehydrogenase
	gi302577543	Clocel_1811	formate acetyltransferase
	gi302578645	Clocel_2965	chaperonin GroEL
	gi302578533	Clocel_2849	calcium binding hemolysin protein
	gi302579527	Clocel_3873	glutamine synthetase
	gi302577047	Clocel_1305	LPXTG-motif cell wall anchor domain-containing protein
	gi302576042	Clocel_0272	hypothetical protein Clocel_0272
	gi302578850	Clocel_3176	Ig domain-containing protein
	gi302575815	Clocel_0038	extracellular solute-binding protein
	gi302578161	Clocel_2460	LPXTG-motif cell wall anchor domain-containing protein
	gi302578736	Clocel_3058	acetyl-CoA acetyltransferase
	gi302577157	Clocel_1416	chaperone protein DnaK
	gi302578534	Clocel_2850	hemolysin-type calcium-binding protein
	gi302575885	Clocel_0108	NLP/P60 protein
	gi302578652	Clocel_2972	3-hydroxyacyl-CoA dehydrogenase
	gi302576470	Clocel_0711	basic membrane lipoprotein
	gi302579442	Clocel_3784	Formate--tetrahydrofolate ligase
	gi302575964	Clocel_0192	acetyl-CoA acetyltransferase
	gi302576478	Clocel_0719	glyceraldehyde-3-phosphate dehydrogenase, type I
	gi302576955	Clocel_1211	Ig domain-containing protein
	gi302576928	Clocel_1184	fibronectin type III domain-containing protein
	gi302577301	Clocel_1565	trigger factor
	gi302576273	Clocel_0510	Heat shock protein Hsp90-like
	gi302576480	Clocel_0721	triosephosphate isomerase
	gi302579460	Clocel_3802	PpiC-type peptidyl-prolyl cis-trans isomerase
	gi302578197	Clocel_2496	YhgE/Pip C-terminal domain-containing protein, partial
	gi302577067	Clocel_1326	branched-chain amino acid aminotransferase

gi302577781	Clocel_2050	hypothetical protein Clocel_2050
gi302579747	Clocel_4097	hydrogenase, Fe-only
gi302577509	Clocel_1777	ribosome recycling factor
gi302577066	Clocel_1325	ketol-acid reductoisomerase
gi302577916	Clocel_2187	hypothetical protein Clocel_2187
gi302576147	Clocel_0379	rubrerythrin
gi302577917	Clocel_2188	hypothetical protein Clocel_2188
gi302579753	Clocel_4103	extracellular ligand-binding receptor
gi302577451	Clocel_1719	Flagellar hook-length control protein-like, C-terminal domain, partial
gi302577440	Clocel_1708	flagellar hook-associated 2 domain-containing protein
gi302577064	Clocel_1323	dihydroxy-acid dehydratase
gi302577507	Clocel_1775	translation elongation factor Ts
gi302578760	Clocel_3082	CTP synthase
gi302577433	Clocel_1701	flagellar hook-associated protein FlgK
gi302577689	Clocel_1957	valyl-tRNA synthetase
gi302578449	Clocel_2764	Penicillin-binding protein dimerization domain
gi302578656	Clocel_2976	Enoyl-CoA hydratase/isomerase
gi302579333	Clocel_3675	phosphate butyryltransferase
gi302578875	Clocel_3201	extracellular solute-binding protein
gi302577713	Clocel_1981	dihydrodipicolinate synthase
gi302577392	Clocel_1660	H+transporting two-sector ATPase alpha/beta subunit
gi302578670	Clocel_2990	dipeptidase
gi302577099	Clocel_1358	family 5 extracellular solute-binding protein
gi302577610	Clocel_1878	serine/threonine protein kinase with PASTA sensor(s)
gi302577853	Clocel_2122	Peptidase M16C associated domain-containing protein
gi302576399	Clocel_0640	fibronectin type III domain-containing protein
gi302578654	Clocel_2974	electron transfer flavoprotein subunit alpha/beta
gi302579370	Clocel_3712	adenylate kinase
gi302576105	Clocel_0337	phospho-2-dehydro-3-deoxyheptonate aldolase
gi302577300	Clocel_1564	hypothetical protein Clocel_1564
gi302579567	Clocel_3913	periplasmic binding protein
gi302578294	Clocel_2596	sugar ABC transporter periplasmic protein
gi302579578	Clocel_3924	histidinol dehydrogenase
gi302578497	Clocel_2812	thiamine biosynthesis protein ThiC
gi302577818	Clocel_2087	hypothetical protein Clocel_2087
gi302579759	Clocel_4109	Ig domain-containing protein
gi302577135	Clocel_1394	LPXTG-motif cell wall anchor domain-containing protein
gi302579400	Clocel_3742	50S ribosomal protein L7/L12
gi302577754	Clocel_2023	hypothetical protein Clocel_2023
gi302577846	Clocel_2115	dTDP-4-dehydrorhamnose reductase
gi302579459	Clocel_3801	PpiC-type peptidyl-prolyl cis-trans isomerase
gi302577202	Clocel_1461	hypothetical protein Clocel_1461
gi302578419	Clocel_2734	pyrroline-5-carboxylate reductase
gi302579423	Clocel_3765	Rfamily protein
gi302576101	Clocel_0333	chorismate mutase
gi302576256	Clocel_0493	dihydroxy-acid dehydratase
gi302576329	Clocel_0570	cellulosome anchoring protein cohesin subunit
gi302576924	Clocel_1180	hypothetical protein Clocel_1180
gi302578429	Clocel_2744	endoribonuclease L-PSP
gi302578079	Clocel_2375	DJ-1 family protein
gi302576998	Clocel_1255	class II aldolase/adducin family protein
gi302579439	Clocel_3781	transcription elongation factor GreA
gi302576999	Clocel_1256	transaldolase
gi302578444	Clocel_2759	beta-lactamase

gi302578718	Clocel_3040	S-adenosylmethionine synthetase
gi302577275	Clocel_1539	RNA binding S1 domain-containing protein
gi302577393	Clocel_1661	H+transporting two-sector ATPase alpha/beta subunit
gi302577063	Clocel_1322	3-isopropylmalate dehydrogenase
gi302579131	Clocel_3465	hypothetical protein Clocel_3465
gi302578737	Clocel_3059	UDP-N-acetylglucosamine 2-epimerase
gi302579079	Clocel_3412	67 kDa myosin-cross-reactive antigen family protein
gi302575853	Clocel_0076	PTS system fructose subfamily transporter subunit IIC
gi302577156	Clocel_1415	GrpE protein HSP-70 cofactor
gi302576350	Clocel_0591	transaldolase
gi302576313	Clocel_0552	hypothetical protein Clocel_0552
gi302577897	Clocel_2166	nicotinate-nucleotide pyrophosphorylase
gi302578452	Clocel_2767	rod shape-determining protein MreC
gi302576479	Clocel_0720	phosphoglycerate kinase
gi302579689	Clocel_4038	hypothetical protein Clocel_4038
gi302578653	Clocel_2973	electron transfer flavoprotein subunit alpha/beta
gi302578329	Clocel_2637	3-oxoacyl-ACP synthase
gi302577242	Clocel_1504	lactaldehyde reductase
gi302578642	Clocel_2962	inosine-5\'-monophosphate dehydrogenase
gi302579344	Clocel_3686	phosphoenolpyruvate-protein phosphotransferase
gi302577137	Clocel_1396	hypothetical protein Clocel_1396
gi302579541	Clocel_3887	ATPase P
gi302579398	Clocel_3740	DNA-directed RNA polymerase subunit beta\'
gi302576117	Clocel_0349	isoleucyl-tRNA synthetase
gi302578646	Clocel_2966	chaperonin Cpn10
gi302576765	Clocel_1013	Ig domain-containing protein
gi302577612	Clocel_1880	ribulose-phosphate 3-epimerase
gi302578084	Clocel_2380	flagellin domain-containing protein
gi302579802	Clocel_4153	rubrerythrin
gi302578856	Clocel_3182	hypothetical protein Clocel_3182
gi302579960	Clocel_4315	uridine phosphorylase
gi302576935	Clocel_1191	extracellular ligand-binding receptor
gi302578878	Clocel_3204	G-D-S-L family lipolytic protein
gi302575781	Clocel_0002	DNA polymerase III subunit beta
gi302577454	Clocel_1722	fagellar hook-basal body protein
gi302579580	Clocel_3926	histidyl-tRNA synthetase 2
gi302576437	Clocel_0678	methyl-accepting chemotaxis sensory transducer
gi302575888	Clocel_0111	peptidase S1 and S6 chymotrypsin/Hap
gi302579558	Clocel_3904	aldo/keto reductase
gi302579867	Clocel_4218	putative GntR family transcriptional regulator
gi302576276	Clocel_0513	extracellular solute-binding protein
gi302579717	Clocel_4067	extracellular solute-binding protein
gi302576344	Clocel_0585	hypothetical protein Clocel_0585
gi302577054	Clocel_1312	argininosuccinate lyase
gi302576078	Clocel_0310	Ig domain-containing protein
gi302577923	Clocel_2194	cellulosome anchoring protein cohesin subunit
gi302578599	Clocel_2917	oligoendopeptidase F
gi302579467	Clocel_3809	SpoVG family protein
gi302579803	Clocel_4154	desulfoferrodoxin
gi302578568	Clocel_2885	cold-shock DNA-binding domain-containing protein
gi302579822	Clocel_4173	acetolactate synthase small subunit
gi302578701	Clocel_3023	dTDP-4-dehydrorhamnose 3,5-epimerase
gi302576315	Clocel_0554	carbonic anhydrase
gi302578977	Clocel_3308	phenazine biosynthesis protein PhzF family

gi302577128	Clocel_1387	hypothetical protein Clocel_1387
gi302576749	Clocel_0997	SpoOM family protein
gi302577452	Clocel_1720	flagellar hook capping protein
gi302579840	Clocel_4191	NADPH-dependent FMN reductase
gi302575935	Clocel_0159	hypothetical protein Clocel_0159
gi302579149	Clocel_3484	phosphoribosyltransferase
gi302579120	Clocel_3454	peptidase M23
gi302577012	Clocel_1269	transketolase
gi302578866	Clocel_3192	hypothetical protein Clocel_3192
gi302578526	Clocel_2842	peptidase M42 family protein
gi302579393	Clocel_3735	translation elongation factor Tu
gi302576390	Clocel_0631	aspartyl aminopeptidase
gi302576077	Clocel_0309	Kelch repeat type 1-containing protein
gi302577767	Clocel_2036	Aluminium resistance family protein
gi302575918	Clocel_0141	NLP/P60 protein
gi302579673	Clocel_4022	carbohydrate kinase
gi302577446	Clocel_1714	flagellar M-ring protein FliF
gi302575921	Clocel_0144	secretion protein HlyD family protein
gi302579399	Clocel_3741	DNA-directed RNA polymerase subunit beta
gi302578183	Clocel_2482	amino acid adenylation domain-containing protein
gi302579549	Clocel_3895	hypothetical protein Clocel_3895
gi302579455	Clocel_3797	histone family protein DNA-binding protein
gi302577458	Clocel_1726	flagellar basal body-associated protein FliL
gi302577103	Clocel_1362	cupin
gi302577061	Clocel_1320	acetolactate synthase small subunit
gi302579391	Clocel_3733	50S ribosomal protein L3
gi302577125	Clocel_1384	hypothetical protein Clocel_1384
gi302575893	Clocel_0116	hypothetical protein Clocel_0116
gi302577772	Clocel_2041	purine nucleoside phosphorylase I
gi302577471	Clocel_1739	fagellar hook-basal body protein
gi302577302	Clocel_1566	ATP-dependent Clp protease, proteolytic subunit ClpP
gi302579401	Clocel_3743	50S ribosomal protein L10
gi302577586	Clocel_1854	phosphate binding protein
gi302579402	Clocel_3744	50S ribosomal protein L1
gi302578988	Clocel_3320	hypothetical protein Clocel_3320
gi302577496	Clocel_1764	MTA/SAH nucleosidase
gi302577324	Clocel_1589	family 3 extracellular solute-binding protein
gi302576466	Clocel_0707	hypothetical protein Clocel_0707
gi302578170	Clocel_2469	isocitrate dehydrogenase
gi302579362	Clocel_3704	DNA-directed RNA polymerase subunit alpha
gi302577969	Clocel_2241	acetyl esterase
gi302578378	Clocel_2690	pyridoxine biosynthesis protein
gi302578584	Clocel_2901	phosphofructokinase
gi302578854	Clocel_3180	leucyl aminopeptidase
gi302579470	Clocel_3812	class I and II aminotransferase
gi302577406	Clocel_1674	peptidase M18 aminopeptidase I
gi302578729	Clocel_3051	ATP synthase F1 subunit alpha
gi302576092	Clocel_0324	hypothetical protein Clocel_0324
gi302578855	Clocel_3181	M6 family metalloprotease domain-containing protein
gi302578351	Clocel_2663	cell wall hydrolase/autolysin
gi302578463	Clocel_2778	PTS system glucose-specific transporter subunit IIBC
gi302578524	Clocel_2840	pyruvate ferredoxin/flavodoxin oxidoreductase
gi302578562	Clocel_2879	hypothetical protein Clocel_2879
gi302576494	Clocel_0735	hypothetical protein Clocel_0735

gi302579383	Clocel_3725	50S ribosomal protein L29
gi302575886	Clocel_0109	transcriptional regulator, AbrB family
gi302579792	Clocel_4143	phosphopantetheine-binding protein
gi302577819	Clocel_2088	ACT domain-containing protein
gi302577183	Clocel_1442	histidine triad (HIT) protein
gi302576832	Clocel_1085	dinitrogenase iron-molybdenum cofactor biosynthesis protein
gi302579855	Clocel_4206	guanine-specific ribonuclease N1 and T1
gi302577432	Clocel_1700	FlgN family protein
gi302577548	Clocel_1816	transcription elongation factor GreA
gi302579450	Clocel_3792	RNA binding S1 domain-containing protein
gi302576314	Clocel_0553	fructose-1,6-bisphosphate aldolase
gi302579403	Clocel_3745	50S ribosomal protein L11
gi302578040	Clocel_2333	hypothetical protein Clocel_2333
gi302578298	Clocel_2601	aconitate hydratase domain-containing protein
gi302578862	Clocel_3188	heat shock protein DnaJ domain-containing protein
gi302576200	Clocel_0435	family 3 extracellular solute-binding protein
gi302577389	Clocel_1657	H+transporting two-sector ATPase E subunit
gi302576030	Clocel_0258	cof family hydrolase
gi302577544	Clocel_1812	pyruvate formate-lyase activating enzyme
gi302576678	Clocel_0920	hypothetical protein Clocel_0920
gi302577675	Clocel_1943	sporulation transcriptional activator Spo0A
gi302577442	Clocel_1710	flagellin domain-containing protein
gi302578826	Clocel_3151	pyridoxal-5\'-phosphate-dependent protein subunit beta
gi302577988	Clocel_2262	short-chain dehydrogenase/reductase SDR
gi302577599	Clocel_1867	YicC-like domain-containing protein
gi302577935	Clocel_2207	porphobilinogen deaminase
gi302577652	Clocel_1920	peptidase M24
gi302579496	Clocel_3838	periplasmic binding protein/LacI transcriptional regulator
gi302577126	Clocel_1385	hypothetical protein Clocel_1385
gi302579477	Clocel_3819	peptidase M15B and M15C DD-carboxypeptidase VanY/endolysin
gi302578655	Clocel_2975	acyl-CoA dehydrogenase domain-containing protein
gi302578411	Clocel_2726	phosphatidylinositol-specific phospholipase C X region
gi302578795	Clocel_3117	hemolysin-type calcium-binding protein
gi302579466	Clocel_3808	UDP-N-acetylglucosamine pyrophosphorylase
gi302578872	Clocel_3198	N-acylglucosamine 2-epimerase
gi302578918	Clocel_3244	peptidase domain-containing protein
gi302579697	Clocel_4046	N-acetyl-gamma-glutamyl-phosphate reductase
gi302579696	Clocel_4045	arginine biosynthesis bifunctional protein ArgJ
gi302578531	Clocel_2847	secretion protein HlyD family protein
gi302577792	Clocel_2061	metal dependent phosphohydrolase
gi302576764	Clocel_1012	Ig domain-containing protein
gi302576947	Clocel_1203	flavodoxin/nitric oxide synthase
gi302579443	Clocel_3785	ATP-dependent metalloprotease FtsH
gi302578963	Clocel_3293	leucyl-tRNA synthetase
gi302577425	Clocel_1693	CheA signal transduction histidine kinase
gi302579418	Clocel_3760	ATPase AAA-2 domain-containing protein
gi302577049	Clocel_1307	hypothetical protein Clocel_1307
gi302577758	Clocel_2027	amino acid adenylation domain-containing protein
gi302578322	Clocel_2630	phosphopantetheine-binding protein
gi302579331	Clocel_3673	TrpR-like protein YerC/YecD
gi302578561	Clocel_2878	phosphotransferase system PTS lactose/cellobiose-specific IIA subunit
gi302577725	Clocel_1993	hypothetical protein Clocel_1993
gi302576387	Clocel_0628	hypothetical protein Clocel_0628
gi302577413	Clocel_1681	hypothetical protein Clocel_1681

gi302578041	Clocel_2334	hypothetical protein Clocel_2334
gi302579392	Clocel_3734	30S ribosomal protein S10
gi302578564	Clocel_2881	PTS system lactose/cellobiose-specific transporter subunit IIB
gi302579572	Clocel_3918	phosphoribosyl-ATP diphosphatase
gi302578740	Clocel_3062	RpiB/LacA/LacB family sugar-phosphate isomerase
gi302577737	Clocel_2006	translation initiation factor IF-3
gi302577444	Clocel_1712	flagellar basal-body rod protein FlgC
gi302578420	Clocel_2735	nitrogen regulatory protein P-II
gi302579529	Clocel_3875	hypothetical protein Clocel_3875
gi302580006	Clocel_4361	50S ribosomal protein L9
gi302577710	Clocel_1978	2,3,4,5-tetrahydropyridine-2,6-carboxylate N-succinyltransferase
gi302577985	Clocel_2258	2-dehydro-3-deoxyphosphogluconate aldolase
gi302577428	Clocel_1696	CheW protein
gi302580011	Clocel_4367	single-strand binding protein
gi302575931	Clocel_0155	zinc-binding CMP/dCMP deaminase
gi302579979	Clocel_4334	SEC-C motif domain-containing protein
gi302576272	Clocel_0509	Pfpi family intracellular protease
gi302578707	Clocel_3029	peptidase M22 glycoprotease
gi302579809	Clocel_4160	hypothetical protein Clocel_4160
gi302579546	Clocel_3892	interferon-induced transmembrane protein
gi302575958	Clocel_0185	hypothetical protein Clocel_0185
gi302577470	Clocel_1738	fagellar hook-basal body protein
gi302577933	Clocel_2205	porphobilinogen synthase
gi302579575	Clocel_3921	phosphoribosylformimino-5-aminoimidazole carboxamide ribotide isomerase
gi302577430	Clocel_1698	CheC, inhibitor of MCP methylation / FliN fusion protein
gi302577895	Clocel_2164	quinolinate synthetase complex subunit A
gi302579428	Clocel_3770	3-methyl-2-oxobutanoate hydroxymethyltransferase
gi302577288	Clocel_1552	HtrA2 peptidase
gi302577193	Clocel_1452	hypothetical protein Clocel_1452
gi302578978	Clocel_3309	CheW domain-containing protein
gi302579932	Clocel_4285	Endonuclease I
gi302579306	Clocel_3648	methyl-accepting chemotaxis sensory transducer
gi302577760	Clocel_2029	phosphoserine aminotransferase
gi302576729	Clocel_0975	hypothetical protein Clocel_0975
gi302578727	Clocel_3049	ATP synthase F1 subunit beta
gi302575900	Clocel_0123	OmpA/MotB domain-containing protein
gi302577806	Clocel_2075	protein-export membrane protein SecD
gi302577017	Clocel_1274	carboxyl-terminal protease
gi302575792	Clocel_0013	seryl-tRNA synthetase
gi302578430	Clocel_2745	cell envelope-related transcriptional attenuator
gi302577851	Clocel_2120	phosphoglucomutase/phosphomannomutase alpha/beta/alpha domain I
gi302576198	Clocel_0433	hypothetical protein Clocel_0433
gi302576157	Clocel_0389	pyruvate kinase
gi302579438	Clocel_3780	lysyl-tRNA synthetase
gi302575865	Clocel_0088	hypothetical protein Clocel_0088
gi302576954	Clocel_1210	Ig domain-containing protein, partial
gi302578812	Clocel_3135	hypothetical protein Clocel_3135
gi302579761	Clocel_4112	peptidase S8 and S53 subtilisin kexin sedolisin
gi302577101	Clocel_1360	hypothetical protein Clocel_1360
gi302575920	Clocel_0143	bacteriocin ABC transporter
gi302579599	Clocel_3945	amino acid adenylation domain-containing protein
