# Supplementary figures and images for: Exoproteome analysis of Clostridium cellulovorans in natural soft-biomass degradation
Source: AMB Express. 2015 Jan 24;5:2. doi: 10.1186/s13568-014-0089-9 (PMC4305082; doi:10.1186/s13568-014-0089-9)

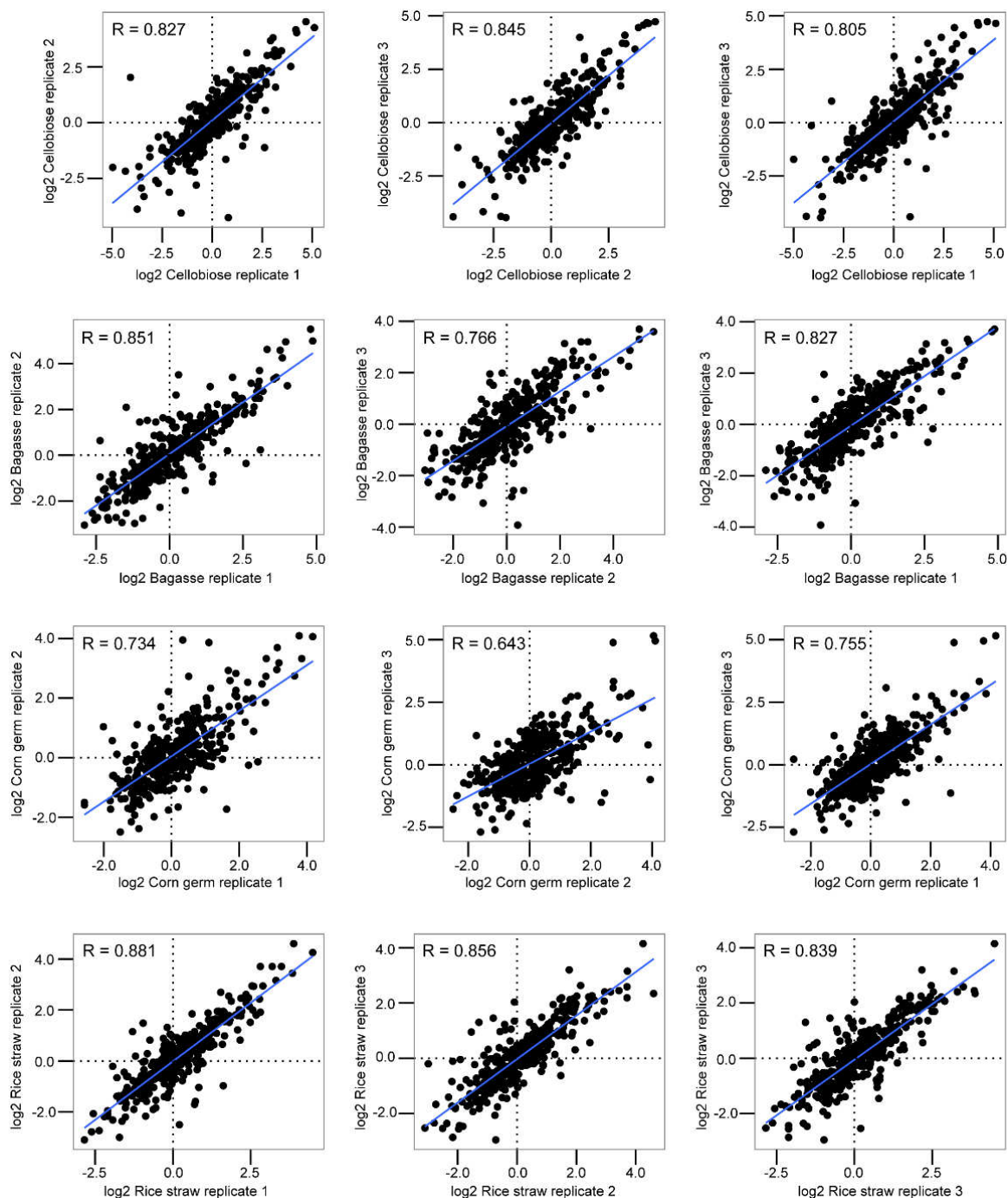

Additional file 2 Esaka et al.

Supplement: Additional file 2: — Scatter plots of the three biological replicates for each substrate. The fold-change values of identified 372 proteins (Additional file 1) by using the Reporter Ions Quantifier with the TMT 6-plex method were normalized using global median. Scatter plots of normalized values were depicted using the data derived from three biological replicates of each culture (cellobiose, bagasse, corn germ, and rice straw). The values of Pearson’s correlation were successfully high in each combination. [file 13568_2014_89_MOESM2_ESM.pdf]

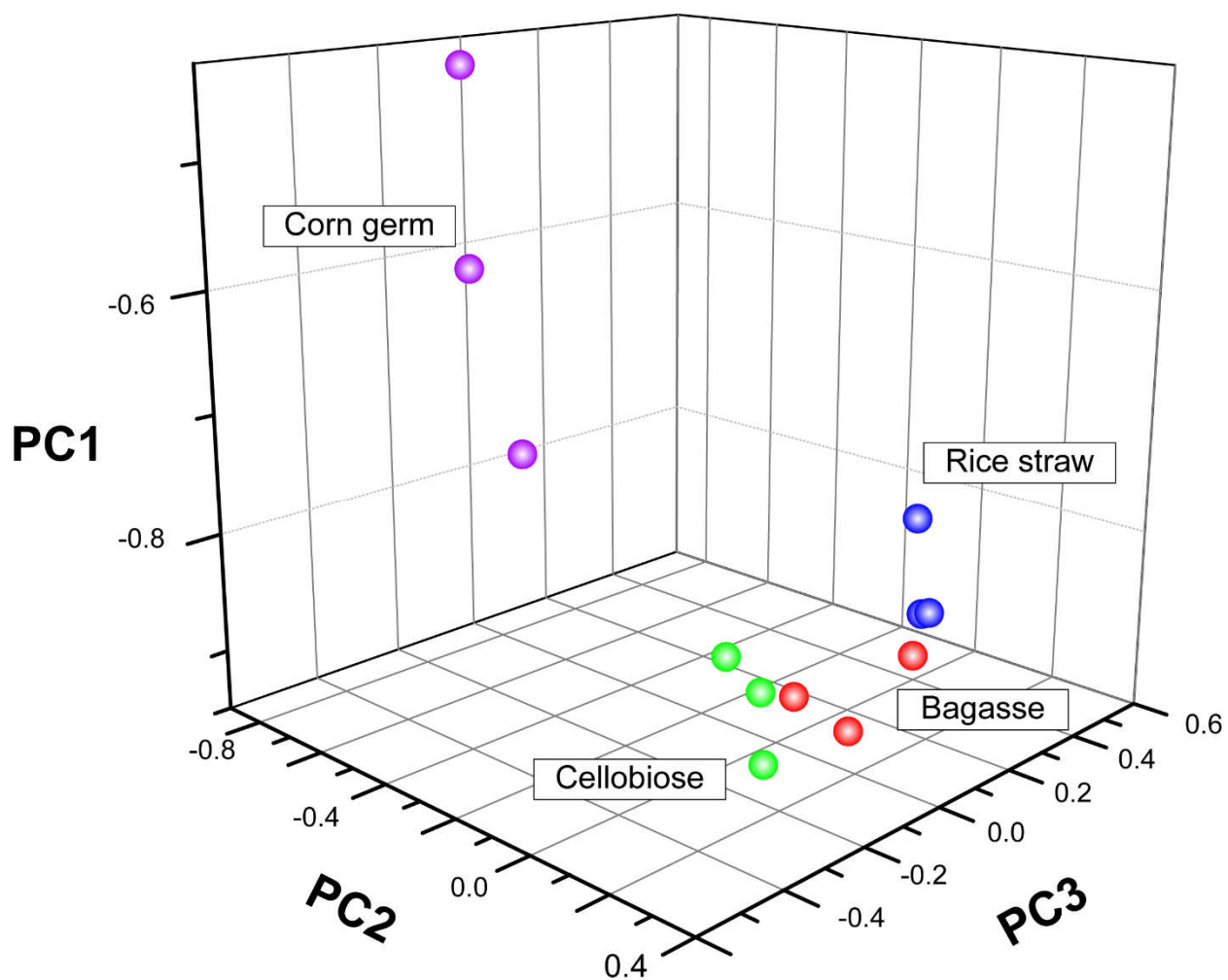

Additional file 3 Esaka et al.

Supplement: Additional file 3: — Principal component analysis of the data from the three biological replicates. Principal component analysis was performed using normalized fold-change values of identified 372 proteins (Additional file 1) for investigation of the similarity of protein production profile between each biological replicate. Proteome data from each substrate clustered in close proximity. The cumulative contribution rate for principal component (PC) PC1 to PC3 was 87.5% (green: cellobiose; red: bagasse; purple: corn germ; blue: rice straw). [file 13568_2014_89_MOESM3_ESM.pdf]
